# Use of a Synthetic Biosensor for Neutralizing Activity-Biased Selection of Monoclonal Antibodies against Atroxlysin-I, an Hemorrhagic Metalloproteinase from *Bothrops atrox* Snake Venom

**DOI:** 10.1371/journal.pntd.0002826

**Published:** 2014-04-24

**Authors:** Francisco Santos Schneider, Dung Le Nguyen, Karen Larissa Castro, Sandra Cobo, Ricardo Andrez Machado de Avila, Nivia de Assis Ferreira, Eladio Flores Sanchez, Christophe Nguyen, Claude Granier, Pascale Galéa, Carlos Chávez-Olortegui, Franck Molina

**Affiliations:** 1 Departamento de Bioquímica e Imunologia, Instituto Ciências Biológicas, Universidade Federal de Minas Gerais, Belo Horizonte, Brasil; 2 SysDiag, UMR3145,CNRS/BioRad, Montpellier, France; 3 Departamento de Pesquisa e Desenvolvimento, Fundação Ezequiel Dias, Belo Horizonte, Brasil; Universidad de Costa Rica, Costa Rica

## Abstract

**Background:**

The snake *Bothrops atrox* is responsible for the majority of envenomings in the northern region of South America. Severe local effects, including hemorrhage, which are mainly caused by snake venom metalloproteinases (SVMPs), are not fully neutralized by conventional serum therapy. Little is known about the immunochemistry of the P-I SVMPs since few monoclonal antibodies (mAbs) against these molecules have been obtained. In addition, producing toxin-neutralizing mAbs remains very challenging.

**Methodology/Principal Findings:**

Here, we report on the set-up of a functional screening based on a synthetic peptide used as a biosensor to select neutralizing mAbs against SVMPs and the successful production of neutralizing mAbs against Atroxlysin-I (Atr-I), a P-I SVMP from *B. atrox*. Hybridomas producing supernatants with inhibitory effect against the proteolytic activity of Atr-I towards the FRET peptide Abz-LVEALYQ-EDDnp were selected. Six IgG1 Mabs were obtained (named mAbatr1 to mAbatr6) and also two IgM. mAbatrs1, 2, 3 and 6 were purified. All showed a high specific reactivity, recognizing only Atr-I and *B. atrox* venom in ELISA and a high affinity, showing equilibrium constants in the nM range for Atr-I. These mAbatrs were not able to bind to Atr-I overlapping peptides, suggesting that they recognize conformational epitopes.

**Conclusions/Significance:**

For the first time a functional screening based on a synthetic biosensor was successfully used for the selection of neutralizing mAbs against SVMPs.

## Author Summary

In this work, we propose a new screening strategy to produce monoclonal antibodies against Atr-I, a P-I class SVMP from *Bothrops atrox*, which is the snake responsible for the majority of the accidents in South America. SVMPs are the main toxic factors in Bothrops venom causing systemic and local hemorrhage, which may evolve to inflammation and/or necrosis. Since the toxic effects of SVMPs are related to their proteolytic activity, we have produced a peptide which was used as a biosensor for Atr-I hydrolysis. Hydrolysis of this substrate was monitored and the clones possessing inhibitory activity against the proteolytic activity of Atr-I upon the peptide were selected. Using our new approach, we have obtained four monoclonal antibodies highly specific and with neutralizing capacity against the hemorrhagic activity of either Atr-I alone or *Bothrops atrox* whole venom. To the best of the authors' knowledge, this is the first time where a functional screening is used for the selection of neutralizing mAbs against SVMPs. It is also the first description of mAbs anti-Atr-I, with inhibitory potential against its toxic activities which may be useful for diagnosis and treatment in the future.

## Introduction

Snakebites cause up to 1,800 000 envenomations per year, mainly in tropical areas [1]–[4]. Snakebites might be considered as a daily occupational hazard since rural subsistent farming communities are the main population suffering from this condition [3], [4], considered as a Neglected Tropical Condition by WHO (World Health Organization) since 2008 [1]. In Brazil, nearly 30,000 snakebite envenomings occur per year and the incidence is about 14 cases/100,000 people/year, a number as high as those found in many other Latin American countries [1], [5]–[8]. Moreover, in the Brazilian Amazon region, 9,000 snakebites occur per year with an incidence fourfold higher than that found in the rest of Brazil. *Bothrops atrox* is found in tropical lowlands and rainforests in the north of South America and is responsible for the majority of envenomations in this area, causing approximately 80% of snake bites [8]–[10]. *B. atrox* envenoming is characterized systemically by headache, severe coagulopathy, with consumption of coagulation blood factors, generalized hemorrhage and renal failure. Locally, severe tissue lesions may be observed, including swelling, blisters, inflammatory response, erythema, ecchymosis, local hemorrhage and necrosis [11], [12].

Immunotherapy by antivenoms is the only efficacious treatment approved by WHO for snakebite accidents. Antivenoms are produced by hyper immunization of animals (generally horses, sheeps or goats) with a pool of venoms from the most important species of snakes found in each country/region [13]. It is known that serum therapy is effective against several of the systemic noxious effects of snake envenomings, when administered early enough [14]–[16]. However, the local effects are not fully neutralized, being clinically important [17]–[20] due to complications related to local hemorrhage and tissue necrosis that can permanently provoke a disability and morbidity among patients, causing a very important socio-economic impact [21].

*B. atrox* venom is a rich mixture of bioactive components belonging to few protein families [22]–[24]. Proteomic characterization of toxin composition of *B.* atrox venom used in this study indicates that the main components of this venom are represented by SVMPs (Snake Venom Metalloproteinases) (58.2%), including P-III and P-I classes, SVSP (Snake Venom Serine Proteinases) (11.17%), PLA_2_ (Phospholipase A_2_) (11.0%) and others [25]. Although these molecules act synergistically in a typical “pit viper envenoming” clinical picture, it is well established that SVMPs are responsible for the most severe local effects (i.e. hemorrhage and its variable consequences) [11], [26]–[31]. SVMPs are zinc-dependent proteinases representing up to 70% of venom dry weight, and can be classified into three classes (P-I to P-III) and several subclasses, according to their domain organization [22]–[24], [32]–[35]. The P-I class are endopeptidases possessing the metalloproteinase catalytic domain only. The P-II class presents both metalloproteinase and disintegrin domains and the P-III class SVMPs contain disintegrin-like and cysteine-rich domains, in addition to the proteinase domain.

Although no P-III class SVMPs from *B. atrox* have yet been characterized at the protein level, evidence supporting the presence of this class of enzymes has been provided by proteomic and transcriptomic studies [23]–[25], [36]. In addition, three P-I class SVMPs from *B. atrox* venom have already been purified and characterized [37]–[39]. Atr-I (Atroxlysin-I), Batx-I and Batroxase are P-I enzymes isolated from *B. atrox* venom from the Amazonian regions of Peru, Colombia and Brazil, respectively. They are hemorrhagic and fibrinogenolytic and do not bear any pro-coagulant activity. These molecules are hemorrhagins that can act proteolytically upon extracellular matrix components, contributing to the local damages following bite. They can also play a systemic role, causing myotoxic effects [38] and inhibition of platelet aggregation [37], which can contribute to hemorrhagic, necrotic and blood-clotting disturbances [37]–[39].

Considering the important role played by P-I class SVMPs in the *B. atrox* poisoning, polyclonal and monoclonal antibodies against these molecules constitute highly useful tools to investigate the structural determinants of toxicity, for patient diagnosis and may have great potential for the preparation of more efficient antivenoms for passive immunotherapy or even for vaccination. Thus, in this study we used a functional screening based on a synthetic biosensor to produce neutralizing mAbs against Atr-I. Specificity assays of Atr-I using oxidized insulin B-chain as substrate showed that the enzyme cleaves the Ala_14_-Leu_15_ peptide bond [37]. Inhibition of enzymatic-dependent cleavage of the Atr-I peptide substrate [Abz-LVEALYQ-EDDnp, containing a FRET (Fluorescence Resonance Energy Transfer) system], determined by means of fluorescence emission, was used as a tool to select specific monoclonal antibodies. Four monoclonal antibodies that fully neutralize the proteolytic and hemorrhagic *in vivo* activities of Atr-I and crude *B. atrox* venom were obtained using this functional screening.

## Methods

### Ethics statement

This study was performed in accordance with the Guide for the Care and Use of Laboratory Animals published by the US National Institutes of Health (NIH Publication No. 85-23, revised 1996) (A5452-01) and were approved by the Animal Experimentation Ethics Committee of the Universidade Federal de Minas Gerais (License number 200/2010).

### Animals and venoms

Animals were maintained at the Centro de Bioterismo of the Instituto de Ciências Biológicas of the Federal University of Minas Gerais, Brazil and received water and food under controlled environmental conditions.

Venoms pooled from at least 5–6 adult *B. barnetti*, *B. brazili*, *B. castelnaudi*, *B. chloromelas*, *B. hyoprora*, *B. microphthalmus*, *B. peruvianus*, *B. pictus*, *B.taeniata* and Peruvian *B. atrox* specimens were generously donated by the Instituto Nacional de Salud (Lima, Perú). Venom from Brazilian *B. atrox*, *Lachesis muta muta*, *Crotalus durissus* and *Micrurus frontalis* were ceded by Fundação Ezequiel Dias (FUNED – Belo Horizonte, Brazil).

### Atroxlysin-I purification

Atroxlysin-I was purified as described earlier [37], using 1.252 mg of *B. atrox* crude venom collected in the Amazon region of Ucayali – Peru. The purity and the molecular mass of 23 kDa were assessed by SDS-PAGE. BCA Kit (Pierce) was used to determine protein concentration following the manufacturer's instructions.

### Production of monoclonal antibodies

#### Hybridoma production

Atr-I was injected four times subcutaneously in BALB/c mice in order to obtain mice hybridomas. 10 µg of protein were used per dose in complete Freund's adjuvant (Sigma) at the first injection, and incomplete Freund's adjuvant (Sigma) at subsequent inoculations with intervals of 2 weeks between each dose. A booster injection of Atr-I was made 4 weeks after the fourth immunization. Throughout the immunization schedule, mice were bled and the reactivity of immune sera was tested against Atr-I in ELISA assay. Three days after the last injection, spleen cells from hyper immunized mice were fused with Sp2/0 myeloma cells (ATCC).

#### Functional clone selection and biosensor synthesis

The FRET peptide (Abz-LVEALYQ-EDDnp) was used as a functional biosensor. Supernatants from resulting hybridomas were functionally screened by inhibition of FRET peptide (Abz-LVEALYQ-EDDnp) hydrolysis by Atr-I. For the production of Abz-LVEALYQ-EDDnp, a first step consisting of Fmoc-Glu-EDDnp synthesis was manually done according to a slightly modified version of the method described by Csuhai and colleagues [40]. Next, Fmoc-Glu-EDDnp was immobilized to Rink Amide resin (Novabiochem) by its lateral chain. The subsequent steps of deprotection and coupling of the aminoacids (Abz-LVEALY) were automatically performed by Fmoc chemistry using Multipep robot (Intavis), as previously described [41]. At the end of the synthesis, the rink amide resin liberates a NH_2_ group, transforming the Glu into a Gln from the sequence Abz-LVEALYQ-EDDnp.

Fmoc amino acids were acquired from Novabiochem and Boc-2-Abz-OH from Sigma Aldrich. The release from the resin and side-chain deprotection was achieved by treatment with trifluoroacetic acid. The peptide Abz-LVEALYQ-EDDnp was purified and analyzed by reverse-phase HPLC (Waters) on a C-18 column with an acetonitrile gradient (0–60%) (not shown).

To select hybridomas of interest, 40 µL of supernatants from cell cultures were pre-incubated with 11 ng of Atr-I for 30 min at 37°C. Then, 10 µL of Abz-LVEALYQ-EDDnp were added to reach a 47 mM final concentration (1∶540 molar ratio enzyme∶substrate). The kinetics of FRET peptide hydrolysis was monitored by fluorescence in a Sinergy2 (Biotek) equipment (λ_ex_ = 320 nm and λ_em_ = 420 nm) for 60 min at 37°C. Positive controls were made with Sp2/0 supernatants culture and the blank had no Atr-I added. Clones possessing inhibitory effects against the proteolytic activity of Atr-I upon FRET peptide were chosen for subcloning by single-cell limiting dilutions. A second selection round was done and subclones presenting the highest inhibitory effect upon Abz-LVEALYQ-EDDnp cleavage by Atr-I were selected for mAbs production. To determine isotypes of mAbs we used IsoStrip (Roche) according to manufacturer's instructions and purified on a protein A-sepharose column (GE Healthcare). The purity of mAbs was analyzed by a 4%–15% gradient SDS-PAGE (Sodium Dodecyl Sulfate-Polyacrylamide Gel Electrophoresis) in non-reducing conditions.

### Molecular characterization of monoclonal antibodies

#### Indirect ELISA

Purified mAbs were tested against several antigens. Maxisorp plates (Nunc) were coated overnight at 4°C with a 1 µg/mL solution of either Atr-I, BaP1 (from *B. asper*), Leucurolysin-a (Leuc-a, P-I from *B. leucurus* venom), Mutalysin-II (Mut-II, P-I from *Lachesis muta muta* venom) or various bothropic crude venoms (i.e. *B. atrox from* Brazil and Peru, *B. barnetti*, *B. brazili*, *B. castelnaudi*, *B. chloromelas*, *B. hyoprora*, *B. microphthalmus*, *B. peruvianus*, *B. pictus*, *B. taeniata* and *Lachesis muta muta)* in a PBS buffer, pH 7.4, and blocked with PBS-Tween 0.1% containing milk (10 g/L). Antibody binding was detected by horseradish peroxidase conjugated donkey anti-mouse whole-IgG (Jackson ImmunoResearch), followed by addition of TMB solution (Bio-Rad). Washes between steps were done in a Tecan microplate washer. Absorbance values were determined at 450 nm with a Tecan Infinite microplate reader.

#### Sandwich-ELISA for differentiation between *B. atrox* and L. *muta muta* venom antigens

Pre-immune sera were obtained from female Swiss mice. Maxisorp plates (Nunc) were coated overnight at 4°C with a solution 1∶100 of anti-bothropic antivenom (FUNED – Belo Horizonte, Brazil) in coating buffer, pH 9.6, and blocked with PBS-Tween 0.1% containing milk (10 g/L). Then, blocked plates were incubated for 60 min at 37°C with several venoms (i.e. Peruvian *B. atrox*, *Lachesis muta muta*, *Crotalus durissus* and *Micrurus frontalis*) diluted at 10 µg/mL in a solution PBS∶mice pre-immune serum (1∶1 vol∶vol) containing Tween 0.1% and milk (1 g/L). After washing, a mixture of mAbatr1, 2, 3 and 6 (5 µg/mL each) was added to plates. Antibody binding was detected by goat anti-mouse IgG antibody, Peroxidase Conjugated (Milipore) followed by addition of OPD Peroxidase substrate (SIGMAFAST from Sigma-Aldrich). Absorbance values were determined at 450 nm. Positive control was performed using *B. atrox* venom at the same concentration as described above diluted in PBS-Tween 0.1% containing milk (1 g/L). Absorbance from wells containing only PBS∶mice pre-immune serum (1∶1 vol∶vol)-Tween 0.1% and milk (1 g/L) were considered as blank values. To be considered as recognized by mAbatrs, a sample should present an absorbance at least double that obtained from the blank, which was chosen as the threshold signal. Multiple Comparisons versus the *B. atrox* group were carried out using One Way ANOVA applying Holm-Sidak method.

#### Immunoassay with cellulose-bound peptides

Overlapping pentadecapeptides frameshifted by 3 residues or octopeptides frameshifted by 1 residue covering the entire amino acid sequence of Atr-I were synthesized by Spot synthesis on cellulose membranes, as previously described [42]. A Multipep (Intavis) robot was used for automated peptide synthesis. After an overnight saturation step with 3% BSA (Bovine Serum Albumin), the set of membrane bound peptides was probed by incubation with mAbs anti-Atr-I (1 µg/mL). Antibody binding was detected by using the alkaline–phosphatase conjugated-goat anti-mouse whole IgG (Sigma, diluted 1∶2000; 90 min, 37°C). After washing, a phosphatase substrate 5-bromo-4-chloro-3-indolyl phosphate (Sigma) and 3-(4,5-dimethylthiazol-2-yl)-2,5 diphenyltetrazolium bromide (Sigma) was added. Anti-Atr-I IgG produced in rabbits were used as positive controls for the membrane [37]. A blue precipitate was observed on peptides bound by antibodies. To allow the re-use of the membranes, they were sequentially treated with dimethylformamide, then 1% SDS, 0.1% 2-mercaptoethanol in 8 M urea, followed by ethanol/water/acetic acid (50∶40∶10 vol/vol/vol) wash and, finally, ethanol so as to remove the precipitated dye and molecules bound to the peptides.

#### Western blotting

For western blotting, 30 µg of Peruvian *B. atrox* venom were subjected to SDS-PAGE (12%) in non-reducing conditions. The proteins were transferred onto nitrocellulose membranes and blocked with PBS-Tween 0.3% containing 2% casein. The membranes were incubated with mAbatr1, 2, 3 and 6 (50 µg/mL) for one hour at room temperature. Immunoreactive proteins were detected using anti-mouse IgG conjugated with peroxidase (1∶3000) from Sigma. After washing three times for 5 minutes with PBS-Tween 0.05%, blots were developed using DAB/chloronaphthol, according to the manufacturer's instructions.

#### Kinetic interactions between mAbs and Atr-I

Surface Plasmon Resonance (SPR) was used in a ProteOn (Biorad) system to measure association (k_a_), dissociation (k_d_) and equilibrium (K_D_) constants for Atr-I binding to mAbs. Experiments were made following the manufacturer's protocol. Briefly, a sensor chip was activated by a combination of sulfo-NHS and EDC (Ethylene Dichloride). Then, 50 µg of each mAb were dissolved in a 10 mM acetate buffer (pH 4.5) and covalently immobilized on the sensor chip. The chip surface was blocked with ethanolamine 1 M (pH 8.5). Interactions were analyzed at room temperature with different concentrations of Atr-I (200; 100; 50; 25 and 12.5 nM) in PBS buffer (pH 7.5) injected with a flow rate of 30 µL/min.

### Neutralizing assays

#### *In vitro* neutralizing assay

Purified mAbs were tested for their neutralizing activities upon Abz-LVEALYQ-EDDnp hydrolysis by Atr-I or *B. atrox* whole venom. 60 ng of *B. atrox* venom or 11 ng of Atr-I were pre-incubated with 2 µg of mAbs (molar ratio of 1∶28 – Atr-I∶antibody) for 30 min at 37°C. Then, the substrate was added in a final concentration of 47 mM. Positive controls were done by pre-incubating Atr-I or *B. atrox* crude venom alone for 30 min at 37°C. The residual activity and neutralizing activity were normalized to the positive control. Enzymatic activity was measured as described above.

#### *In vivo* neutralizing assay

Anti-Atr-I mAbs were tested against *B. atrox* venom or Atr-I hemorrhagic activity in Swiss mice as described earlier [43]. One MHD (Minimum Hemorrhagic Dose) of Atr-I (19 µg) [37] or 1.8 MHD of *B. atrox* whole venom (13 µg) [44] was pre-incubated with several amounts of mAbs (12.5; 25.0; 50.0 and 100.0 µg at molar ratios of 10∶1, 5∶1, 2.5∶1 and 1.25∶1 – purified Atr-I∶antibody; or 2.95∶1, 1.48∶1, 0.73∶1 and 0.37 – estimated quantity [37] of Atr-I in *B. atrox* whole venom: antibody) for 1 hour at 37°C in a final volume of 100 µL. The mixture was inoculated subcutaneously in mice. Alternatively, monoclonal antibodies anti-Atr-I (50 or 100 µg) were subcutaneously injected in PBS solution (100 µL) in mice either before or after experimental envenoming with 1.8 MHD of *B. atrox* venom. In both cases, three hours after Atr-I or venom injection the animals were euthanized in a CO_2_ chamber and their skins were removed for evaluation of residual hemorrhage.

### List of accession numbers

Atroxlysin-I (UniProtKB/SwissProt P85420); BaP1 (UniProtKB/SwissProt P83512); Batroxstatin-1 (UniProtKB/SwissProt C5H5D2); Batroxstatin-2 (UniProtKB/SwissProt C5H5D3); Batroxstatin-3 (UniProtKB/SwissProt C5H5D4); Batx-I (UniProtKB/SwissProt P0DJE1); *B. atrox* myotoxin I (UniProtKB/SwissProt Q6JK69); Leucurolysin-a (UniProtKB/SwissProt P84907); Mutalysin-II (formely named LHF-II – UniProtKB/SwissProt P22796) were used/cited in this work.

## Results

### Production and molecular characterization of mAbs

**Figure 1:**
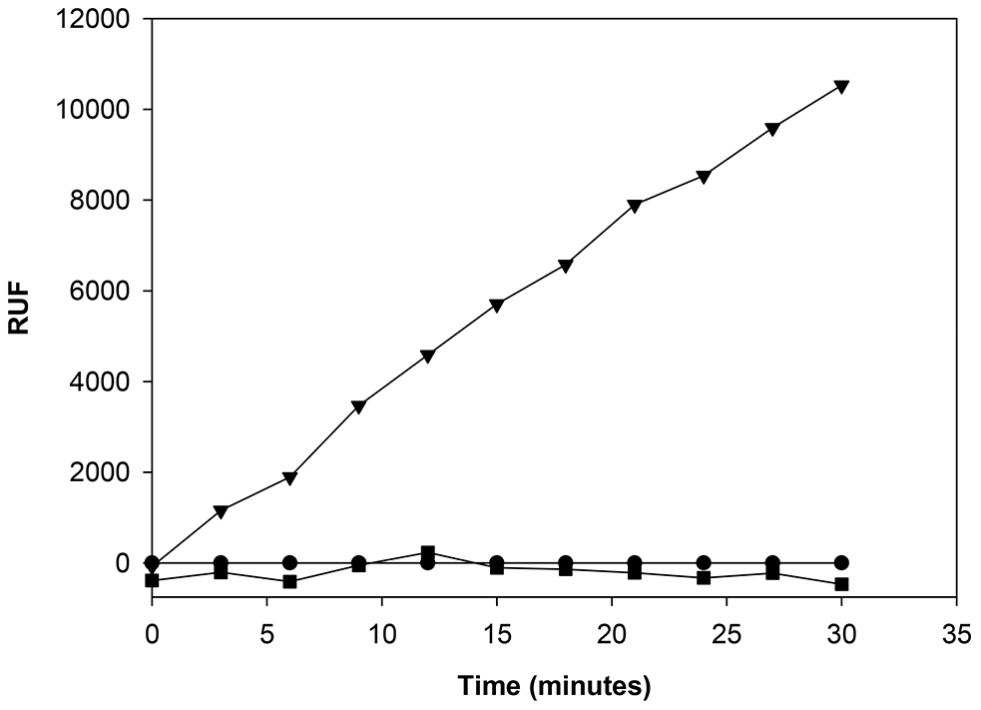
Enzymatic hydrolysis of Abz-LVEALYQ-EDDnp by Atr-I. Cleavage of Abz-LVEALYQ-EDDnp by Atr-I results in a fluorescent emission dependent of the Atr-I enzymatic activity. Hydrolysis of the substrate was assessed after incubation of 11 ng of Atr-I alone (▾) or mixed with EDTA 2 mM (▪) with the FRET substrate for 30 minutes at 37°C. Abz-LVEALYQ-EDDnp alone was used as negative control (•).

To try to bias the selection of mAbs towards antibodies with Atr-I neutralizing activity, we devised an hybridoma screening assay based on the capacity of hybridoma supernatants to block the proteolytic activity of Atr-I towards the synthetic substrate Abz-LVEALYQ-EDDnp (Figure 1).

**Figure 2:**
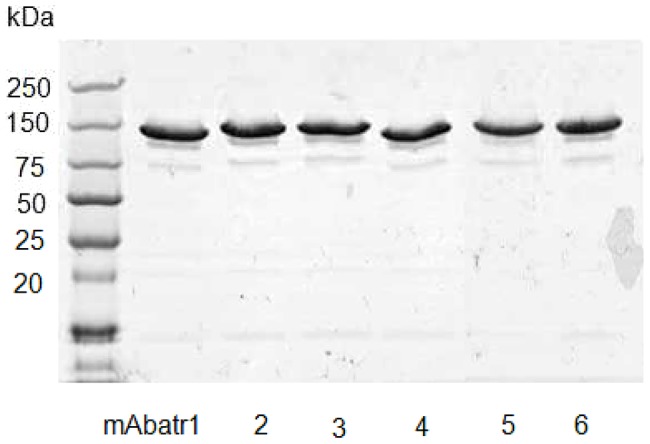
SDS-PAGE of purified mAbatrs. Six anti-Atr-I monoclonal antibodies, isotyped as IgG1, were purified on a protein A-Sepharose column. They appear as homogeneous fractions on SDS-PAGE using a gradient gel (4–15%) in non-reducing conditions.

After immunization of BALB/c mice with Atr-I, a panel of twenty-one anti-Atr-I secreting hybridomas was selected on the basis of their capacity to neutralize Abz-LVEALYQ-EDDnp hydrolysis by Atr-I. Only hybridomas that presented at least 50% of inhibition of the Atr-I proteolytic activity were selected. These clones were then subcloned and a new round of selection was performed. Eight clones presenting the highest inhibition of the proteolytic activity of Atr-I were finally chosen for production, of which six were IgG1 (named mAbatr1 to mAbatr6) and two were IgM. mAbatr1 to 6 were purified on a protein A-Sepharose column and appeared as homogeneous bands on SDS-PAGE (Figure 2).

**Figure 3:**
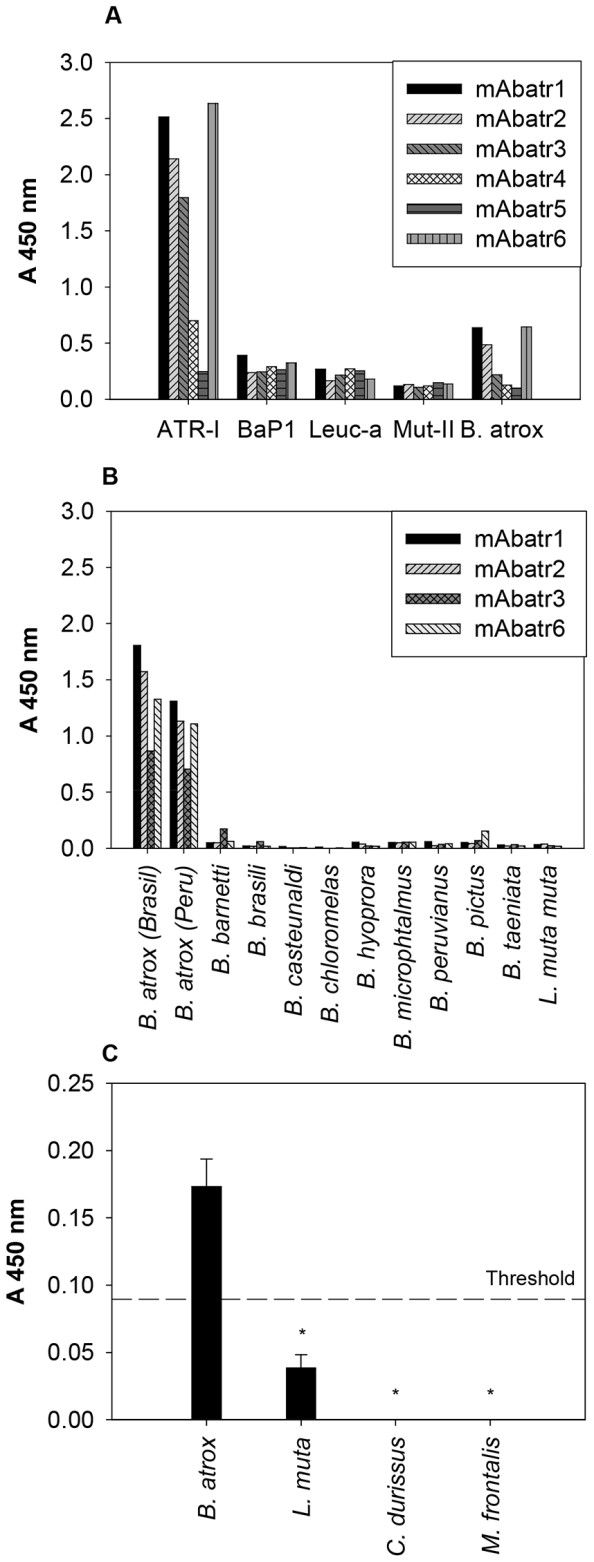
Antigenic reactivity of selected monoclonal antibodies (mAbatrs). (A) Reactivity of purified mAbatrs (5 µg/mL) against several P-I SVMPs and *B. atrox* venom and BSA (negative control-not shown) was measured by ELISA. (B) Reactivity of mAbatr1, 2, 3 and 6 against *B. atrox* (from Brazil and Peru), *B. barnetti*, *B. brasili*, *B. casteunaldi*, *B. chloromelas*, *B. hyoprora*, *B. microphtalmus*, *B. peruvianus*, *B. pictus*, *B. taeniata* e *Lachesis muta muta* venoms (40 µg/mL) was accessed by ELISA. (C) Reactivity of polled mAbatr1, 2, 3 and 6 in sandwich ELISA against Peruvian *B. atrox* venom, *L. muta muta*, *C. durissus* and *M. frontalis* venoms (10 µg/mL) diluted in mice sera simulating experimental envenoming. Positive controls were performed using diluting *B. atrox* venom at the same concentration diluted in a PBS buffer (not shown). Threshold absorbance is represented as at least double that obtained from the blank wells. (*p<0.001). Results are expressed as mean of the absorbance value of triplicates.

The eventual cross-reactivity of the selected mAbatrs with several P-I SVMPs (i.e. Atr-I, BaP1, Leuc-a and Mut-II) and *B. atrox* crude venom was tested in an ELISA format. Figure 3A shows that mAbatrs were not able to recognize the heterologous P-I SVMPs tested. On the other hand, mAbatr1, 2, 3 and 6 presented a high reactivity against Atr-I and a moderate reactivity with *B. atrox* whole venom. mAbatr4 weakly recognized Atr-I and presented low reactivity against the crude venom, while mAbatr5 reacted neither with Atr-I nor *B. atrox* venom. mAbatr1, 2, 3 and 6 were also tested against several South American snake venom antigens (Figure 3B). All mAbs tested showed high specific reactivity against *B. atrox* venom in our ELISA conditions. The other venoms used as antigens coated to ELISA plates were not recognized by the mAbatrs.

Based on the specificity of mAbatrs1, 2, 3 and 6, we decided to evaluate their potential application as diagnostic tools for *B. atrox* simulating experimental envenoming. In sandwich ELISA using plates coated with polyspecific anti-bothropic antivenom from FUNED (Brasil), mAbatrs recognized *B. atrox* venom exclusively with an absorbance signal significantly higher (p<0.001) compared to all other venoms tested (Figure 3C).

**Figure 4:**
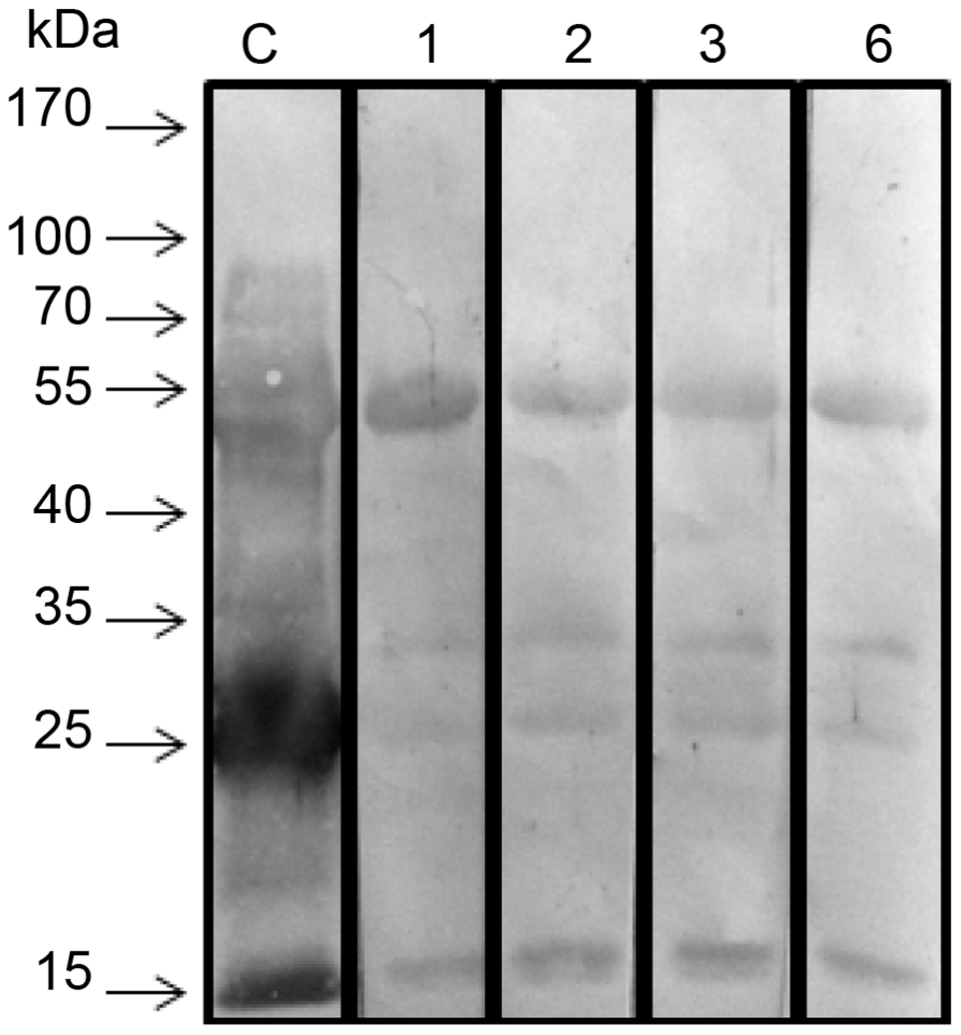
Cross-reactivity of mAbatrs with different toxins from *B. atrox* venom analyzed by western blotting. *B. atrox* crude venom was transferred to a nitrocellulose membrane and incubated with rabbit polyclonal anti-Atr-I serum (**C**) as control, or mAbatr1 (**1**), mAbatr2 (**2**), mAbatr3 (**3**) or mAbatr6 (**6**). All mAbatrs recognized bands around 55, 30, 23 and 15 kDa.

mAbatr1, 2, 3 and 6 were also tested against *B. atrox* venom in Western Blot. All mAbatrs tested recognized four bands around 15, 23, 30 and 55 kDa (Figure 4).

**Figure 5:**
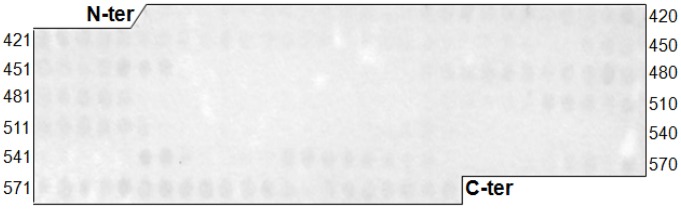
Molecular pattern of mAbatrs recognition. Reactivity of 8-mer overlapping peptides derived from the amino acid sequence of Atr-I. Peptides were prepared by the Spot method on cellulose membranes and binding of mAbatrs (1 µg/mL) to cellulose-bound peptides was detected by an alkaline phosphatase-conjugated anti-mouse antibody (diluted 1∶1000). None of mAbatrs showed reactivity against linear sequence of Atr-I.

In an attempt to understand mAbatrs epitope recognition on Atr-I, octa- and pentadecapeptides frameshifted by 1 or 3 residues, respectively, covering the amino acid sequence of Atr-I were synthesized by the SPOT technique and tested either with mAbatr1, 2, 3 and 6 (Figure 5) or IgG anti-Atr-I from rabbit as positive control. None of the mAbatrs was capable of reacting with the linear peptides covering the Atr-I primary sequence. However, rabbit anti-Atr-I polyclonal IgGs exhibited reactivity against linear epitopes of Atr-I (not shown - manuscript in preparation), suggesting that epitope recognition by mAbs requires folding of Atr-I into its native structure.

**Figure 6:**
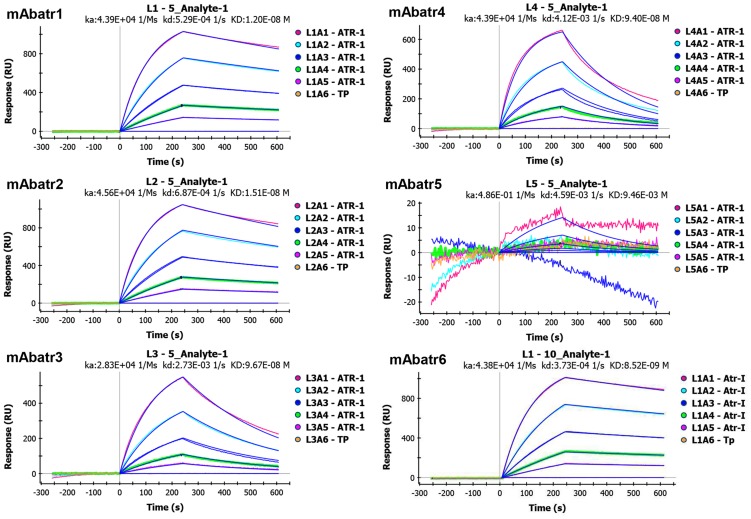
mAbatrs affinity to Atr-I. Kinetic parameters were measured in a ProteOn system. mAbatrs were immobilized on a chip and different concentrations of Atr-I were injected in a flow of 30 µL/min. Binding was evaluated at room temperature.

The kinetic parameters of mAbatrs interaction with Atr-I were measured on a ProteOn system (BioRad). This equipment measures up to 36 interactions simultaneously, allowing comparisons of affinity among mAbatrs, since they are tested at the same time. Association (k_a_), dissociation (k_d_) and equilibrium (K_D_) constants for Atr-I binding to mAbs are shown in figure 6. mAbatr1, 2 and 6 showed high affinity to Atr-I with equilibrium constants in the 10^−9^ M range, whilst mAbatr3 and mAbatr4 showed slightly lower affinities (K_D_ = 9.67×10^−8^ M and 9.40×10^−8^ M). mAbatr5 was not able to react with Atr-I, corroborating the ELISA's results. The interaction of BaP1, Leucurolysin-a and Mutalysin-II with mAbatr1, 2, 3 and 6 were also tested on ProteOn, but no binding was detected (not shown).

### Neutralizing assays

**Figure 7:**
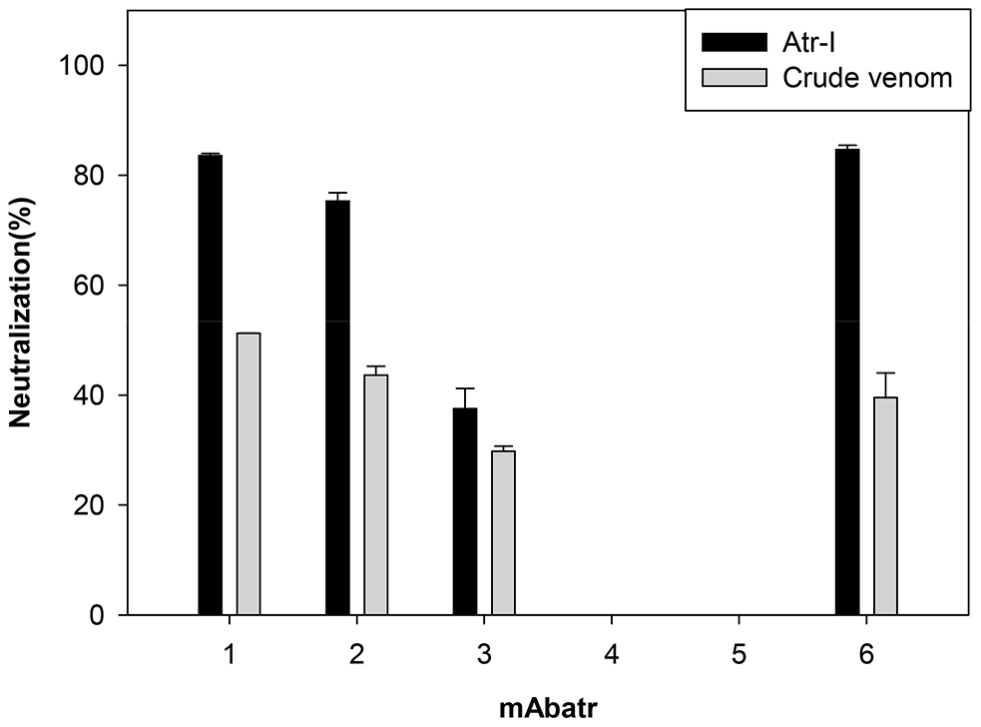
Neutralization of Atr-I or *B. atrox* venom enzymatic activities on Abz-LVEALYQ-EDDnp substrate. Purified mAbatrs were pre-incubated with Atr-I or *B. atrox* venom at 37°C for 30 minutes previously to addition of the FRET substrate. Results are normalized to Atr-I or *B. atrox* venom alone (positive control) and represent means ±S.D. of triplicates.

The mAbatrs were tested both *in vitro* and *in vivo* in order to assess their neutralizing ability against purified Atr-I or *B. atrox* whole venom. Inhibition of the proteolytic activity of Atr-I or *B. atrox* crude venom on Abz-LVEALYQ-EDDnp substrate cleavage is shown in figure 7. mAbatr1 and 6 exhibited around 85% neutralization of the maximal effect (mAbatr1 – 83.56%±0.40; mAbatr6 – 84.67%±0.78). mAbatr2 showed a weaker blocking of Atr-I activity (75.30%±1.5) and mAbatr-3 only demonstrated a moderate neutralizing effect (37.55%±3.67). When tested against *B. atrox* whole venom *in vitro*, mAbatr1, 2, 3 and 6 presented a weaker neutralization ability (51.24%±0.01; 43.63%±1.62; 29.79%±0.93 and 39.58%±4.45, respectively). On the other hand, mAbatr4 and 5 did not neutralize enzymatic proteolysis of the synthetic substrate induced by Atr-I in the tested conditions.

**Figure 8:**
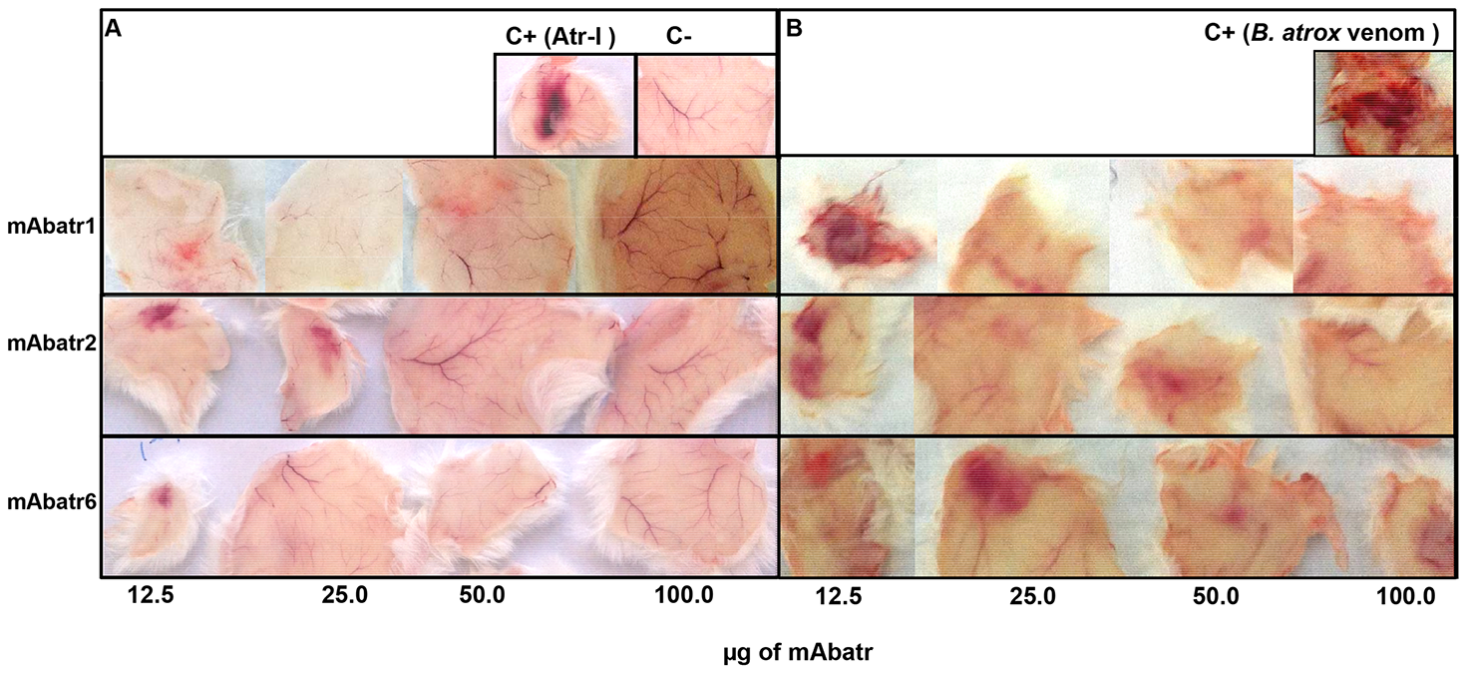
Neutralization of Atr-I-induced hemorrhage in mice. Residual hemorrhage was evaluated after incubating mAbatr1, mAbatr2 or mAbatr3 with 1 MHD of Atr-I (**A**) or 1.8 MHD of *B. atrox* crude venom (**B**) and injecting subcutaneously in mice. After 3 hours mice were euthanized and skins removed. Negative control was saline and positive controls were Atr-I or *B. atrox* venom alone.

To reduce testing in living animals, only the three strongest *in vitro* neutralizing antibodies (mAbatr1, 2 and 6) were tested against Atr-I or *B. atrox* venom induced hemorrhage. One MHD of Atr-I was pre-incubated with mAbatrs at different concentrations and then injected subcutaneously in mice. mAbatr1 and 6 fully neutralized the hemorrhagic activity induced by atr-I at a molar ratio of 5∶1 (Atr-I : mAbatr), while mAbatr2 only neutralized the hemorrhage at a 2.5∶1 molar ratio or higher (Atr-I : mAbatr2) (Figure 8A). 1.8 MHD of *B. atrox* crude venom was completely neutralized by mAbatr1 at 50 µg (0.73∶1 molar ratio Atr-I: mAbatr1) or higher, while mAbatr2 and 6 inhibited *B. atrox* hemorrhagic activity at 100 µg (0.37∶1 molar ratio Atr-I: mAbatr) (figure 8B).

However, when injected without preincubation with *B. atrox* venom, none of the mAbatrs was able to neutralize hemorrhage induced by *B. atrox* venom at any dose tested (not shown).

## Discussion

The bothropic envenoming induces severe local symptoms in human victims, including hemorrhage and tissue necrosis. Complications arise in up to 40% of the cases, in which permanent damage can necessitate the amputation of limbs. The local noxious effects of bothropic venoms (e.g. hemorrhage and necrosis) are mainly due to the action of SVMPs [11], [26]–[30], which seem to be not well neutralized by current therapeutic anti-bothropic antivenoms [17]–[20]. In this present work, we describe a new rational and functional method to produce neutralizing monoclonal antibodies against P-I SVMPS, using as hybridoma selection criterion the capability of mAbs to block the proteolysis induced by SVMPs.

Atr-I, a 22.3 kDa P-I class SVMP isolated from the Peruvian *B. atrox* venom, is able to hydrolyze the peptide bond between ala_14_ and leu_15_ present in insulin B-chain [37], as well in the fluorogenic peptide (Abz- LVEALYQ-EDDnp). We decided to synthesize this peptide sequence coupled to a fluorescent donor and its respective quencher and to use the FRET technique to measure the inhibition of Atr-I-induced hydrolysis of this biosensor by mAbs. Hybridoma supernatants abolishing fluorescent emission were selected as potentially neutralizing mAbs. Based on this method, we have obtained six IgG1 and two IgM monoclonal antibodies against Atr-I.

mAbs previously produced against BaP1 or Mut-II were selected by ELISA and presented cross-reactivity against heterologous venoms and P-I class SVMPs [45]–[47]. Although our mAbatrs were not selected on an antigen-binding capacity basis, they showed a very high specificity to Atr-I and *B. atrox* venom. We demonstrated that they do not recognize either other P-I SVMPs from Latin American pit viper venoms that present high degrees of similarity to Atr-I (i.e. Mut-II and BaP1, 57% of identity and Leuc-a, 52% of identity), or any other South American whole venoms from species that share the Amazonia forest as habitat with *B. atrox*.

In the Amazonian forest, the snake *Lachesis muta muta* is responsible for approximately 10% of all snakebites, and the symptoms of this accident are very similar to *B. atrox* envenoming. Currently, there is no laboratory diagnostic able to differentiate *B. atrox* from *L. muta muta* envenoming. Therefore, we have developed a simple test for discriminating *B. atrox* envenoming from envenoming caused by other genera. To avoid unnecessary animal suffering, we have bled Swiss mice and prepared a mixture of their sera with different venoms, simulating experimental envenoming. ELISA plates were coated with polyspecific anti-bothropic antivenom (FUNED – Brasil) to capture antigens from different venoms. Using a pool of mAbatrs, only antigens from *B. atrox* venom were recognized, presenting an absorbance signal double that of pre-immune sera, suggesting that our mAbatrs could be useful in the development of a differential diagnostic for *B. atrox* envenoming.

Western blot assays using mAbatr and purified Atr-I or *B. atrox* venom were done under reducing (not shown) and non-reducing conditions. Under reducing conditions, no reactivity was observed. In non-reducing conditions, mAbatr1, 2, 3 and 6 recognized four bands at approximately 15, 23, 30 and 55 kDa. Earlier works have shown the presence of several toxins in *B. atrox* venom [23], [24], [48], [49], including: *B. atrox* myotoxin I, a secreted Lys49 PLA_2_ with an calculated MW of 13,826 [50], which possesses 37% of identity compared to Atr-I; a P-I SVMP named Batroxase [39], which contains 90% of identical residues compared to Atr-I; as well as three SVMPs from P-III class, called batroxstatin-1, -2 and -3, which possess up to 60% of identity at the proteinase domain compared to Atr-I [36], [39]. Due to the likely evolution of toxins by gene duplication and diversification, some epitopes may be kept unchanged during evolution [24], [51], [52] and common epitopic motifs recognized by mAbatrs might be shared by some different classes of SVMPs in *B. atrox* venom, which could explain the cross-reacting bands around 30 and 55 kDa in Western Blot assay, as observed before [46], [47], [53], [54]. However, the recognition of the band at ∼15 kDa is probably not the result of an interaction between mAbatrs and PLA_2_ since the overall shape of PLA_2_ molecules differ from SVMPs structures. Thus it is reasonable to assume that the reacting band at 15 kDa might be either an artifact or result of SVMPs autolysis/degradation.

None of our mAbatrs recognized overlapping synthetic peptides from the Atr-I primary sequence, confirming that the conformation of Atr-I is very important in recognition by mAbatrs and suggesting that all mAbatrs bind to conformational epitopes. Few works have reported on the molecular interaction of monoclonal antibodies with their respective epitopes in SVMPs. Apparently, neutralizing mAbs against P-I SVMPs interact predominantly via conformational structures [45]–[47]. Further studies are necessary to better characterize the functional epitopes recognized by mAbatrs and to clarify their role in the biological activity of Atr-I. However, recognition of loops adjacent to the methionine-turn near to the catalytic region, which is an important region for the catalytic activity and determines substrate specificity, might explain the neutralizating activities of mAbatrs. Moreover, this region presents the highest variability in SVMPs, which could account to the high mAbatrs' specificity for Atr-I [55], [56].

mAbatr6 (K_D_ = 8.52×10^−9^ M), mAbatr1 (K_D_ = 12.0×10^−9^ M) and mAbatr2 (K_D_ = 15.1×10^−9^ M), showed the highest affinities for Atr-I. Despite the fact that the hybridoma selection method we designed was based on function and not on binding, mAbatrs presented nanomolar equilibrium constants for their binding to Atr-I. This affinity is in the same range as that found in mAbs against others SVMPs, which were selected through affinity-based ELISA assays. When selected conventionally by binding assays, mAbs against SVMPs do not present a correlation between affinity and inhibitory action [45], [47]. However, it is interesting to note that there is a clear correlation between the measured affinity of mAbatrs to Atr-I and their neutralizing efficacy.

Three mAbs (mAbatr1, mAbatr2 and mAbatr6) efficiently neutralized proteolysis induced by both Atr-I and *B. atrox* venom upon Abz-LVEALYQ-EDDnp. As hemorrhagic activity of SVMPs is dependent on their enzymatic activities, we decided to test whether mAbatr1, 2 and 6 could prevent hemorrhage induced by either Atr-I or *B. atrox* venom *in vivo*. Although mAbatr1, mAbatr2 and mAbatr6 presented a slightly weaker inhibitory activity on synthetic substrate compared to anti-BaP1 monoclonal antibodies MABaP1-3 and MABaP1-6; mAbtr1, 2 and 6 fully neutralized *in vivo* hemorrhage induced by Atr-I isolated or *B. atrox* whole venom when preincubations with mAbs were performed.

Brazilian and Peruvian *B. atrox* venoms are composed mainly by SVMPs, including P-I and P-III classes [21]–[25], which are the main molecules responsible for the hemorrhagic activity in *B. atrox* envenoming. The possible cross-reactivity against some other SVMPs present in *B. atrox* venom, but not all of them, may play a key role in neutralization of *B. atrox* whole venom in hemorrhage and could explain the weaker neutralizing ability of mAbatrs against *B. atrox* whole venom tested *in vitro*. The neutralization of other bothropic South American venoms was not tested, since all mAbatrs were very specific and recognized only *B. atrox* venom.

Currently, the standard procedure used, even for monoclonal antibodies, to measure the neutralization capacity of an antivenom against the hemorrhagic activity of snake venoms consists in the preparation of a mixture of venom and antivenom, followed by injection of this preincubated mixture in animals [45], [46], [53], [57], [58]. Thus, it is reasonable to assume that once mAbatrs bind to Atr-I and/or Atr-I-like molecules in preincubation step of our hemorrhagic assay these hemorrhagins are sterically hindered and not able to bind to their *in vivo* molecular targets, leading to the abolishment of hemorrhage. On the other hand, when mAbatrs are not preincubated with *B. atrox* venom, hemorrhage is still observed, suggesting that when hemorrhagins are first injected in animals, they bind to and degrade their *in vivo* molecular targets and become inaccessible to mAbatrs. Further studies are needed to clarify the efficacy of preincubation steps of mabs and SVMPs in studies of neutralization of hemorrhage induced by SVMPs.

In conclusion, we developed an efficient method for functional antibody screening, based on a synthetic biosensor to produce mAbs specifically neutralizing P-I SVMPs *in vitro* and *in vivo*. To the best of our knowledge, this is the first time that a functional screening has been used in order to select monoclonal antibodies able to block the toxic effects of SVMPs. It is also the first description of mAbs against Atr-I, isolated from *B. atrox* venom, with inhibitory potential against toxic activities of purified Atr-I and *B. atrox* crude venom. It is still unknown where neutralizing mAbatrs bind to Atr-I. Further, mAbatrs are highly specific to *B. atrox* antigens and may be useful as diagnostic tools for *B. atrox* envenoming. These very encouraging results open the way for a wider utilization of synthetic biosensors in functional screening aiming at the production of neutralizing monoclonal antibodies for further therapeutic approaches or diagnostic assays against *B. atrox* envenoming.
